# Supplementary material for: Circulating Amino Acids and Risk of Peripheral Artery Disease in the PREDIMED Trial
Source: Int J Mol Sci. 2022 Dec 23;24(1):270. doi: 10.3390/ijms24010270 (PMC9820556; doi:10.3390/ijms24010270)
Supplement: Supplementary file 1 [file ijms-24-00270-s001.zip › ijms-2050439-supplementary.pdf]

Supplementary material

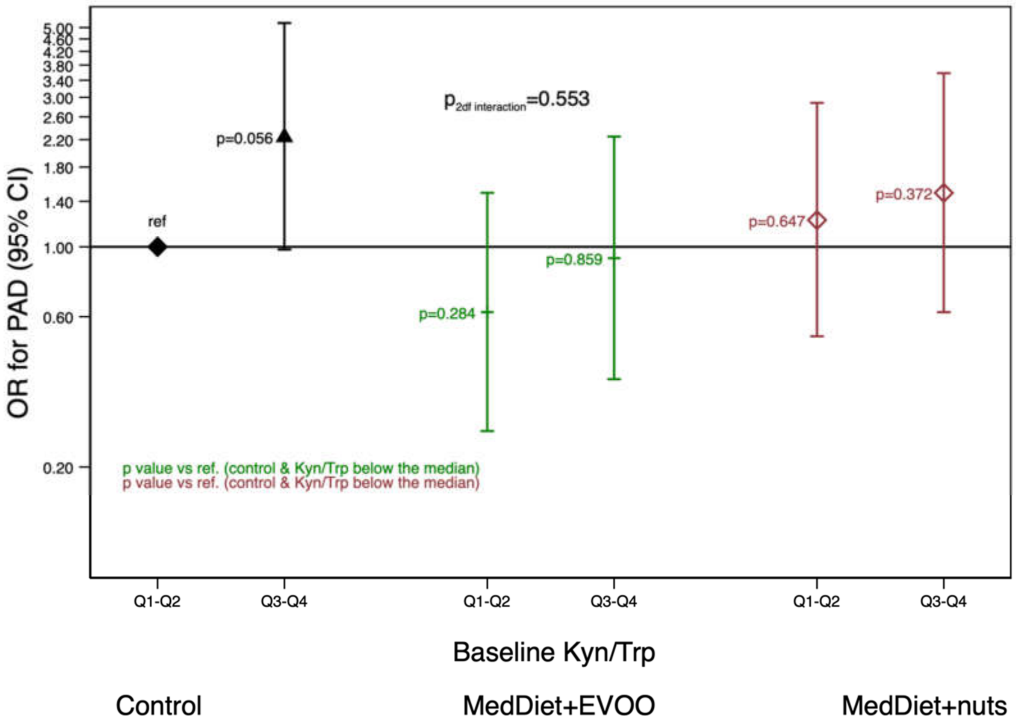

Figure S1. Matched odds ratios (95% CI) for the association between baseline kynurenine to tryptophan ratio (Kyn/Trp) (below/above the median) and incident peripheral artery disease (PAD) within each PREDIMED intervention arm. Multivariable model adjusted for intervention group (Mediterranean diet vs control group), body mass index (kg/m2), smoking (never, current, former), leisure-time physical activity (metabolic equivalent tasks in min/d), prevalent chronic conditions at baseline (dyslipidemia, hypertension and diabetes), educational level (primary or lower/secondary or higher) and propensity scores predicting randomization to account for small between-group imbalances at baseline.

MedDiet: Mediterranean diet

Kyn/Trp: kynurenine to tryptophan ratio

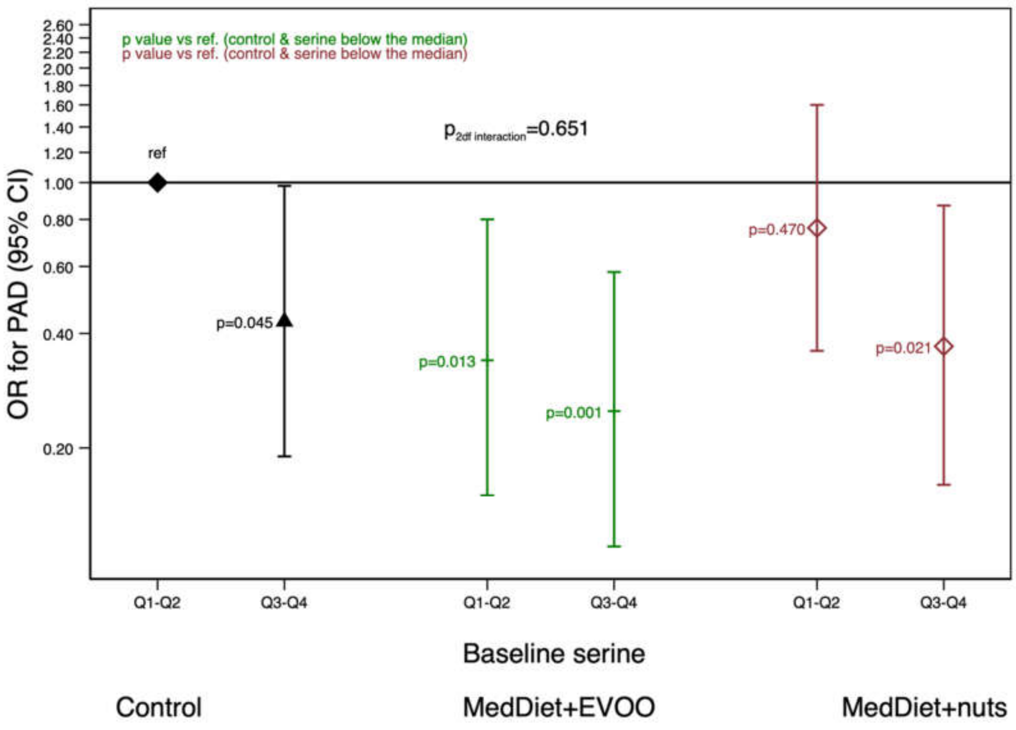

Figure S2. Matched odds ratios (95% CI) for the association between baseline serine (below/above the median) and incident peripheral artery disease (PAD) within each PREDIMED intervention arm. Multivariable model adjusted for intervention group (Mediterranean diet vs control group), body mass index (kg/m2), smoking (never, current, former), leisure-time physical activity (metabolic equivalent tasks in min/d), prevalent chronic conditions at baseline (dyslipidemia, hypertension and diabetes), educational level (primary or lower/secondary or higher) and propensity scores predicting randomization to account for small between-group imbalances at baseline.

MedDiet: Mediterranean diet.
